# Influence of atmosphere and austenitic stainless steel on the solar salt corrosivity

**DOI:** 10.1016/j.heliyon.2024.e25966

**Published:** 2024-02-09

**Authors:** Sumit Kumar, Andrea Hanke, Alexander Bonk, Thomas Bauer

**Affiliations:** a German Aerospace Center (DLR), Institute of Engineering Thermodynamics, 70569, Stuttgart, Germany; b German Aerospace Center (DLR), Institute of Engineering Thermodynamics, 51147, Köln, Germany

**Keywords:** Nitrate salt chemistry, Thermal energy storage (TES), Concentrated solar power (CSP), Gold particle tracing, Corrosion process

## Abstract

The utilization of the Solar Salt (60 wt%NaNO_3_/40 wt%KNO_3_) mixture as a Thermal Energy Storage (TES) medium is gaining importance due to its scalability and cost-effectiveness. However, the corrosion of metallic components presents a significant challenge. This study explores the intricate interplay between salt chemistry and its corrosivity, particularly at elevated temperatures exceeding the state-of-the-art bulk temperature 565 °C. The study manipulates salt decomposition by adjusting the oxygen partial pressure in the purge gas over Solar Salt and investigates the evolution of salt chemistry with and without the presence of steel. It analyzes the corrosion behavior of two types of stainless steel, AISI 316L and AISI 310, under different gas purging atmospheres. Furthermore, it employs a gold particle tracing technique to identify and monitor the formation and growth of the corrosion layer on the steel surface. The results reveal that nitrogen gas purging significantly enhances salt decomposition and its corrosivity over time. The presence of steel also influences salt decomposition depending on the purged gas atmosphere. In a nitrogen atmosphere, the presence of steel can increase the nitrite levels, while an air atmosphere results in an elevated concentration of oxide ions. In air, the AISI 310 alloy shows slightly better performance than AISI 316L. Both alloys experience substantial mass loss in the nitrogen-purged atmosphere. Interestingly, the presence of gold particles within the middle of the corrosion layer in the air purged atmosphere visually illustrates a counter diffusion involving various cations and anions across the corrosion layer.

## 1 Introduction

The increasing demand for renewable energy sources has led to developments in solar energy storage. One of the advancements in this field is the use of Solar Salt as a Thermal Energy Storage (TES) medium. Solar Salt is a mixture of 60 wt% NaNO_3_/40 wt% KNO_3_, with a liquidus temperature of about 250 °C and current maximum operation temperature of 565 °C. This mixture has several advantages, including high density, high heat capacity, low vapor pressure, low cost, and low environmental impact [1,2]. Solar Salt-TES systems are commercially used in conjunction with Concentrated Solar Power (CSP) to store thermal energy for dispatchable electricity generation.

Over the past several decades, there has been extensive research into the decomposition and equilibrium reactions of nitrate-based molten salts, resulting in various decomposition reactions [[3], [4], [5], [6]]. It is widely understood that the first step involves the reversible reduction reaction of nitrate (NO_3_^−^) to nitrite (NO_2_^−^) and the release or uptake of oxygen, as shown in Equation (1). The reaction shifts towards the right side (producing more nitrite) with increasing temperature.

(1) $$NO_{3}^{-}⇄NO_{2}^{-}+\frac{1}{2}O_{2}$$

The equilibrium constant K for the above reaction can be given by Equation (2).

(2) $$K=\left[NO_{2}^{-}\right]{\left(P_{O_{2}}\right)}^{\frac{1}{2}}/\left[NO_{3}^{-}\right]$$

[NO₃^-^] and [NO_2_^−^] represent the concentration of nitrate and nitrite ions respectively and (P_O2_)^1/2^ represents the square root of the oxygen partial pressure. Moreover, as highlighted in Equation (2), the nitrate to nitrite conversion can be increased by lowering the oxygen partial pressure, such as through the use of argon as a purging gas.

As temperatures exceed 500 °C, the nitrite formed in Equation (1) can further decompose into various oxide species (O_2_^−^, O_2_^2−^, O^2−^) and nitrogen oxide gases (N_x_O_y_) [[7], [8], [9]], as shown in Equation (3).

(3) $$NO_{2}^{-}⇄N_{x}O_{y}+O^{2-}$$

At the time of writing of this paper, there is a limited understanding of the kinetics, equilibria (e.g., type of oxide species formed), and underlying mechanisms involved in this reaction. Hence, Equation (3) is a simplified description. Sötz et al. [10] have demonstrated that the concentration of overall oxide ions increases over time in a synthetic air environment at temperatures of 600 °C and 620 °C. When adding nitrogen oxide (NO) to the purge gas above the salt melt, the oxide ions concentrations can be reduced. This observation suggests a dependency of the nitrite/oxide equilibrium in the presence of nitrous gases. This concept has been strengthened by the findings of Steinbrecher et al. [11] who demonstrated the regeneration of previously decomposed Solar Salt through the use of a purge gas containing nitrogen oxides. According to the Lux Flood theory, an increase in oxide ion species results in elevated basicity within molten salt systems. Tzvetkoff et al. [12] summarized that when different molten salt systems come into contact with various metals, they have the potential to produce a range of metal oxide products. The specific type of metal oxide formed depends on the level of basicity of the molten salt in the reaction.

The corrosion of metallic components when exposed to molten nitrate salt at elevated temperatures can present a significant challenge within the CSP-TES systems. Numerous researchers have conducted extensive investigations into the corrosion behavior of different metal alloys, considering various factors such as temperature, exposure duration, salt mixture ratios, and impurity levels, as reviewed by Walczak et al. [13].

Many studies have reported the formation of different corrosion layers on stainless steel in contact with molten Solar Salt. The most commonly observed outermost corrosion layer typically consists of a mixture of hematite (Fe_2_O_3_), magnetite (Fe_3_O_4_), as well as wurstite (FeO) above 570 °C [14]. At temperatures exceeding 600 °C, Bradshaw [15] reported the incorporation of sodium within the iron oxide layer. Picard et al. [16] related the sodium incorporation to the basicity of the molten nitrate/nitrite salt melt and stated that sodium-enriched phases such as NaFeO_2_ and Na_4_Fe_2_O_5_ can form in molten salts at high salt basicity. Beneath the iron oxide layer, several authors have reported the presence of a mixture of chromium oxide (Cr_2_O_3_) and iron chromium oxide (FeCr_2_O_4_) spinel structure [17,18]. The chromium oxide layer is widely recognized for its protective nature, attributed to its capacity to generate a dense and stable layer. However, it can dissolve in Solar Salt as chromate (CrO_4_^2−^) due to its high solubility [19]. Rapp et al. [20] explained the solubility of different metal oxides is related to the oxide ions concentration of the melt. Furthermore, earlier studies have demonstrated a correlation between high oxide ions (O_2_^2−^,O^2−^) concentrations of the nitrate/nitrite salt melt and high corrosion rates [21,22]. While numerous studies have primarily focused on corrosion rates, material performance, and the influence of impurities, the interaction of various salt anions, including nitrate, nitrite, and oxide ions, and their contribution to the formation of distinct corrosion layers has often been overlooked in the existing literature.

The primary objective of the presented work is to obtain a deeper understanding of the intricate relationship between the Solar Salt chemistry and the corrosion behavior of austenitic stainless steel. To gain deeper insights into the corrosion process and the role of different anions (nitrate, nitrite, and oxide ions) in Solar Salt on the formation of corrosion layers and products, it is essential to examine how the Solar Salt chemistry evolves concurrently with the corrosion process. This paper investigates, for the first time, the impact of Solar Salt chemistry on austenitic steel corrosion and how steel influences Solar Salt chemistry under two different atmospheres (nitrogen and synthetic air) at 600 °C for 1000 h. Additionally, this study employs a comprehensive array of post-analysis techniques for both molten salt (including Ion Chromatography, titration, and Atomic Absorption Spectroscopy) and steel (SEM-EDX, descaling, and gold particle tracing). Furthermore, reference measurements were conducted in the absence of steel to analyze the influence of steel corrosion on the Solar Salt chemistry. Overall, this study provides a unique aspect of not only the impact of Solar Salt chemistry on corrosion but also *vice versa* the impact of corrosion on the Solar Salt chemistry. This novel systematic approach can significantly contribute to our understanding of the intricate corrosion mechanisms in CSP-TES systems, paving the way for advancements in corrosion mitigation strategies and providing valuable insights into optimizing system performance.

## 2 Materials and methods

### 2.1 Experimental setup

**Fig. 1:**
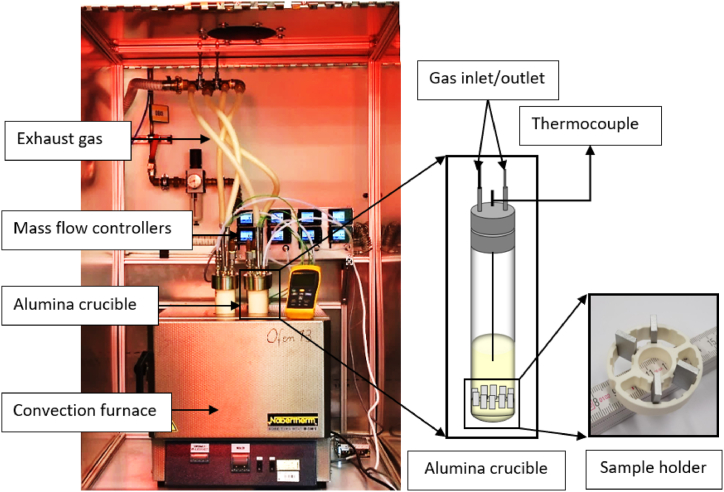
Front view of the modified convection furnace, illustrating the four-alumina crucible on the left and the crucible assembly with the sample holder on the right.

The experiment was conducted using alumina crucibles and a modified convection furnace. The convection furnace was modified by drilling four identical holes for the alumina crucible insertion from the top. Given that Solar Salt decomposes at higher temperatures and has the potential for salt creeping and salt evaporation outside the alumina crucibles during the experiment, there exists the possibility of a consequential weight change. To mitigate the risk of salt creeping, specific precautions were implemented during the experimental setup. The crucibles containing the Solar Salt were positioned to extend approximately 30 mm outside the furnace. A two-part steel flange was employed to maintain a controlled atmosphere within the alumina crucible. The upper part of the flange had ports for gas inlet and outlet. To prevent any potential interaction between Solar Salt and other metallic components, an alumina-based sample holder for steel samples was used. This sample holder was positioned at the bottom of the alumina crucible to ensure full immersion in the molten salt throughout the experiment. Additionally, a K-type thermocouple was deployed to continuously monitor the salt temperature, with the thermocouple being fully immersed in the salt for the duration of the experiment. Fig. 1 illustrates the experimental setup, which is identical to the setup described in detail elsewhere [23].

A total of four alumina crucibles were employed for the experiment. Among these, two crucibles were purged with nitrogen (grade 5.0, Linde gas, Germany) at a flow rate of 100 ml/min, regulated by a digital mass flow controller. The remaining two crucibles were purged with synthetic air (grade 5.0, Linde gas, Germany) at the same flow rate. Within each atmosphere group, one crucible served as the reference, while the other contained the steel samples. The placement of steel samples and atmosphere in the crucibles is indicated in Table 2 based on the respective crucibles.

**Table 1:** Elemental composition in wt.% of the two austenitic steel types according to the material datasheet.

Alloy	C	Cr	Ni	Mo	Mn	Si	P	S	N	Fe
310	≤0.10	24.00–26.00	19.00–22.00	0	≤2.00	≤1.5	≤0.045	≤0.015	≤0.11	Balance
316L	0.03	16.50–18.50	10.00–13.00	2.00–2.50	2	1	0.045	0.015	0	Balance

**Table 2:** Crucible wise sample matrix for steel and salt samples.

Crucible notation	Purge Gas	Steel type	Gold Sputtered Thickness	Steel post analysis	Salt post analysis
C1–N_2_–S	N_2_	AISI 310	4 nm	SEM	IC Titration AAS
		AISI 310		Mass loss by descaling	IC Titration AAS
		AISI 316L	4 nm	SEM	IC Titration AAS
		AISI 316L		Mass loss by descaling	IC Titration AAS
C2-air-S	Air	AISI 310	4 nm	SEM	IC Titration AAS
		AISI 310		Mass loss by descaling	IC Titration AAS
		AISI 316L	4 nm	SEM	IC Titration AAS
		AISI 316L		Mass loss by descaling	IC Titration AAS
C3–N_2-_ref	N_2_	–		–	IC Titration
C4-air-ref	Air	–		–	IC Titration

* SEM: Scanning Electron Microscopy, IC: Ion Chromatography, AAS: Atomic Absorption Spectroscopy.

### 2.2 Experimental procedure

The **Solar Salt** used in this experiment was synthesized using pro analysis grade NaNO_3_ (purity >99.5%, Merck, Germany) and KNO_3_ (purity >99.5%, Merck, Germany) at a 60/40 wt% ratio. For the corrosion experiment, 200 g of the Solar Salt (120 gm NaNO_3_ and 80 gm KNO_3_) mixture were carefully filled into each of the alumina crucibles. Subsequently, all four crucibles were placed inside the furnace and heated to 180 °C overnight to ensure complete removal of any remaining moisture.

The experiment was performed on **two types of steel**: AISI 316L and AISI 310 (see Table 1 for composition).

Before conducting the corrosion test, all austenitic steel samples were prepared by cutting them to a uniform size (20 x 10 × 3 mm), grinding them with SiC paper down to P600 grit, and then thoroughly washing them with deionized water and acetone. Each steel type (AISI 316L and AISI 310) had a total of eight samples, out of which four samples from each steel type were gold sputtered (Q150V ES Plus Quorum) at ultra-high vacuum 10^−6^ mbar for tracing the diffusion layer and products, while the remaining four were used for descaling mass loss.

**Fig. 2:**
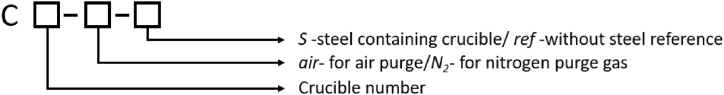
Experiment labeling according to purge gas atmosphere and steel sample.

Four steel samples, comprising two gold-sputtered and two for descaling, of both AISI 316L and AISI 310, were arranged in a single sample holder. Another sample holder was configured in the same manner. These two sample holders were then placed in separate alumina crucibles, each to be purged with a different atmosphere: synthetic air and nitrogen. Additionally, two alumina crucibles are used as a reference experiment without any steel samples for each atmosphere. Table 2 provides details on the specific sample placement and analysis techniques employed for both the salt and steel in each crucible. The crucible labeling is done based on the purge gas atmosphere and the presence of steel, as shown in Fig. 2.

The steel samples were simultaneously extracted from the steel containing crucibles after 500 and 1000 h. The initial salt samples were drawn from all the crucibles at t = 0 h, once the salt temperature reached 600 °C, followed by subsequent sampling at intervals of every 200 and 500 h.

### 2.3 Salt and steel post analysis

The salt samples, collected manually from the four crucibles at intervals of 200 and 500 h and amounting to approximately 2–4 g, were analyzed for nitrate, nitrite, chromate, and oxide ions. Additionally, the salt containing steel was also analyzed for various metallic species. Salt samples were first analyzed using **ion chromatography** (IC) (Metrohm model 880 Basic IC plus) for nitrate, nitrite, and chromate ions. For Ion chromatography 125 mg of salt was dissolved in 500 ml ultra-pure water and analyzed. The detailed description of the experiment and calibration is given elsewhere [24].

Acid-base **titration** (Metrohm Titrando 800, Herisau, Switzerland) was performed to analyze the basicity of the same salt samples by determining the oxide ion concentrations. For titration 500 mg of salt is dissolved in 160 mL ultrapure water and titrated with 0.01 M HCl (Titrisol standard solution) solution. Titer determination was performed five times against sodium carbonate (Merck, Darmstadt, Hessen, Germany, purity >99.5%). Irrespective of the type of oxide ion present in the salt mixture (O^2−^, O_2_^2−^, O_2_^−^), oxide ions are depicted as “O^2−^” in this work. The analysis techniques and statistical analysis titration methods are described elsewhere in detail [11].

To further analyze the presence of any other cations in the salt mixture **Atomic Absorption Spectroscopy** (AAS) was performed using iCE 3000 series atomic adsorption spectrometer from Thermo Fisher Scientific (Waltham, Massachusetts, USA). First, 100–500 mg of salt sample was dissolved with 1 ml of 69% nitric acid with ultrapure water in 50 ml flask. As an ionization buffer, 1 ml of 10% Cesium chloride was added. The AAS was first calibrated using three different standard concentrations of Fe, Cr, and Ni (Roth, certified reference material) ranging from 0.1 to 5 mg/L, and the linear fitting of signal and concentration was done to establish the relation between the observed signal for every metal and sample concentration. Finally, the signal of different metals from the prepared salt solutions was measured and converted from mg/L to wt.% for every element. Thermophysical properties, including melting temperature and thermal stability, were not specifically investigated in this study.

The steel samples were extracted at 500 and 1000 h. To determine the **area-specific mass loss** after corrosion, the descaling methodology described in ASTM G1(C7.4) was employed. The descaling process was repeated for 8 to 10 successive cycles until a steady mass loss was achieved and the mass loss per unit area was calculated according to equation (4).

(4) $$\Delta m=\frac{m_{f}-m_{i}}{S_{0}}$$

Where Δm is the descaled mass loss per unit area ($mg/cm^{2})$, $m_{f}$ is the final sample mass after descaling in (mg), $m_{i}$ is the initial sample mass after descaling in (mg) and $S_{0}$ is the initial sample surface area in ($cm^{2})$.

Additionally, after the corrosion test, the previously gold-sputtered steel samples were cleaned with deionized water, iso-propanol, dried, embedded in silicon resin, polished, and further analyzed by **Secondary Electron Microscopy** (SEM, Zeiss Crossbeam 350). SEM was used to analyze the corrosion layer thickness and **Energy Dispersive X-ray spectroscopy** (EDX) (Oxford instruments) was used for elemental mapping. Both SEM and EDX were the part of same machine setup.

## 3 Results and Discussion

### 3.1 Salt analysis

#### 3.1.1 Salt chemistry without steel

To analyze the influence of atmosphere on Solar Salt chemistry, this subsection specifically examines the evolution of **salt chemistry without steel** under two distinct atmospheres: synthetic air and nitrogen. It focuses on the reference crucibles C3–N_2_-ref and C4-air-ref.

First, the analysis of **nitrate (NO**_**3**_^**−**^**), nitrite (NO**_**2**_^**−**^**), and chromate (CrO**_**4**_^**2−**^**)** content of the two crucibles using IC is discussed. Subsequently, the evaluation of **oxide ion (O**^**2−**^**)** concentration through titration is presented and discussed.

**Fig. 3:**
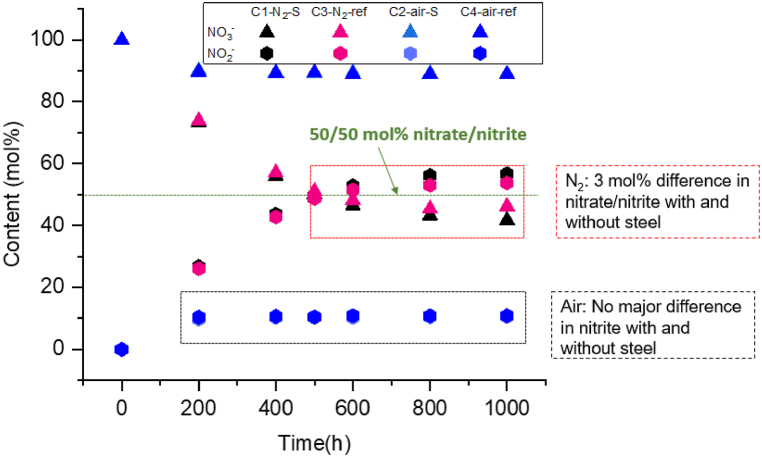
Nitrate and nitrite anion contents in Solar Salt under synthetic air and nitrogen at 600 °C measured by ion chromatography.

Fig. 3 illustrates the **nitrate and nitrite content** in the Solar Salt samples exposed to synthetic air and nitrogen, depicting the results of IC post-analysis over the course of the experiment. In the **synthetic air**-purged crucible (C4-air-ref), the nitrate/nitrite content stabilized after 200 h at 88.8/10.9 mol % and remained stable up to 1000 h, which is in good agreement with the previously reported equilibrium data by Nissen and Meeker (90/10 mol%) [4], or Sötz et al. (91/9 mol%) [10] at an oxygen partial pressure of 0.21 atm and 600 °C. In contrast to the air atmosphere, the nitrate/nitrite content of the Solar Salt samples in the **nitrogen** atmosphere from the crucible (C3–N_2_-ref) exhibited a faster nitrate decomposition rate, reaching a nitrate/nitrite content of 50/50 mol% after 500 h. This behavior can be justified by Equations (1), (2)), as the absence of oxygen in the purged gas is expected to a faster salt decomposition. Subsequently, a slower decomposition rate was observed, resulting in a content of 46.2/53.8 mol% until 1000 h with a very slow increase (quasi-steady-state) of the nitrite content. It was also observed in a study by Bonk et al. [23] under similar conditions but at a lower temperature of 560 °C. Further discussion on this quasi steady-state behavior will follow after exploring the oxide ion formation under air and nitrogen gas purged conditions.

No **chromate ions** were detected in the IC measurements due to the absence of steel in the reference crucibles under both gas-purged conditions. This indicates that there is no other source of chromates other than the steel sample itself.

**Fig. 4:**
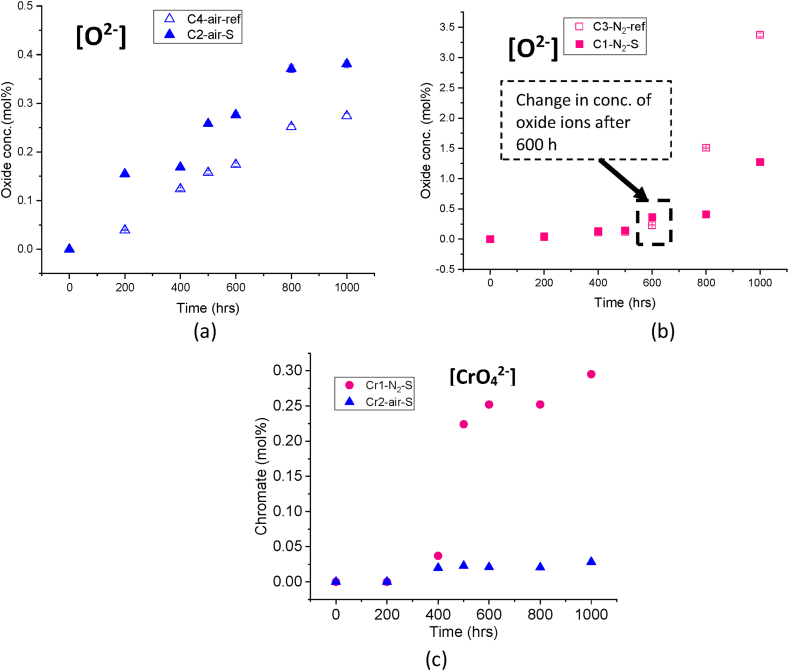
Anion contents in Solar Salt at 600 °C a) Oxide ion concentration in Solar Salt under synthetic air (y-axis range: 0–0.5 mol%) b) Oxide ion concentration in Solar Salt under nitrogen by acid-base titration (y-axis range: 0–3.5 mol%, error bars are within symbol size) c) Chromate content in Solar Salt under two purged atmospheres measured by ion chromatography.

The **oxide ion concentration** determined by titration in the same salt samples is shown in Fig. 4(a) and (b). The air-purged crucibles (C4-air-ref) and nitrogen-purge crucible (C3–N_2_-ref) showed an increasing content of oxide ions over time with final concentrations of 0.27 mol% and 3.37 mol%, respectively. In both cases, these values do not appear to reach a steady-state, most likely due to the absence of nitrogen oxide gases in the purge gas, as indicated by Equation (3). The absence of these gases can push the equilibrium to the right side of the reaction (3) leading to more oxide ion formation over time [10]. In agreement with previous studies under nitrogen atmosphere [25], the increase in the concentration of oxide ions in the nitrogen-purged crucibles is an order of magnitude higher compared to the air-purged crucibles. This can be attributed to the higher salt decomposition, resulting in a higher nitrite content in the nitrogen-purged system. Consequently, this can promote the further formation of oxide ions in the absence of nitrogen oxide gases, as described by Equation (3).

Given the evident quasi-steady-state behavior observed after 500 h in the nitrate/nitrite equilibrium within the nitrogen-purged reference crucible, it becomes apparent, by Equation (2), that the conversion of nitrate to nitrite is influenced by the presence of oxygen partial pressure. In the absence of oxygen, nitrate should fully decompose into nitrite. However, there are several explanations for the quasi-steady-state found in C1–N_2_–S and C3–N_2_-ref. The most likely explanation is, that there is a sufficiently high oxygen partial pressure resulting from the 5.0-purity N_2_ gas flow. This could be attributed to impurities in the purge gas, potential gas leaks in the system, or the presence of a high concentration of oxide ions in the salt, which can alter the nitrate/nitrite equilibrium, as suggested by Paniccia and Zambonin [26]. It's important to mention that this paper does not specifically explore these factors in detail.

#### 3.1.2 Salt chemistry with steel

To analyze the influence of steel on the Solar Salt chemistry, this subsection specifically examines the evolution of **salt chemistry with steel** under two distinct atmospheres: synthetic air and nitrogen. It focuses on the crucibles C1–N_2_–S and C2-air-S. Furthermore, these results are also compared with the reference measurements conducted without steel.

In contrast to the reference crucibles, the crucibles containing steel under both synthetic air and nitrogen purging conditions exhibit distinct behaviors. First, the content of **nitrate, nitrite, and chromate ions** analyzed through IC are discussed. Subsequently, the concentration of **oxide ions** is evaluated through titration. Additionally, AAS measurements are included to determine the concentration of the major alloying elements, namely **Cr, Fe, and Ni**, in the salt containing steel.

As shown in Fig. 3, **the nitrate/nitrite content** in the presence of **steel and synthetic air** purging (C2-air-S), similar to the reference crucible under air purged conditions stabilized at 88.9/10.6 mol%. These findings suggest that the addition of austenitic steel did not significantly alter the equilibrium nitrate/nitrite content in the air-purged crucibles. In the presence of **steel and nitrogen** purge gas (C1–N_2_–S), there is a noticeable increase in salt decomposition, characterized by lower nitrate content and higher nitrite content, specifically 41.7% and 56.7%, respectively. This is in contrast to the reference crucible purged with nitrogen without steel, where nitrate and nitrite concentrations are 46.2% and 53.8%, respectively. The observed difference in the presence of steel indicates the influence of corrosion on altering the salt chemistry. A detailed discussion of this aspect will be provided at the end of the Results and Discussion section (Section 3.2.2), following the analysis of atmosphere impact on the steel corrosion.

The **chromate ions** were detected by IC for the same salt samples and the results are illustrated in Fig. 4(c). In presence of **steel and synthetic air** purging (C2-air-S) the chromate content in the salt increases to 0.028 mol% at the end of 1000 h and appearing to reach a steady state after 500 h. In contrast, the **steel and nitrogen** purge (C1–N_2_–S) exhibit a faster chromate ions formation rate, reaching 0.295 mol% at the end of 1000 h. This concentration is an order of magnitude higher than that observed in the air-purged crucible with steel. Additionally, the chromate content in the nitrogen-purged system with steel did not appear to reach a steady state over 1000 h. The higher concentration of chromate ions observed in the salt from the steel and nitrogen-purged crucible also implies a more pronounced depletion of chromium from the steel in the presence of nitrogen, as compared to crucibles purged with air.

Fig. 4(a) and (b) illustrates the concentration of **oxide ions** in the air- and nitrogen-purged crucibles with steel, respectively. Similar to the oxide ions in the reference air purged crucible, the oxide ions in the **steel and synthetic air** purged crucible (C2-air-S) increases overtime and reaches a value of 0.38 mol%. Interestingly, the presence of steel results in a slightly higher oxide ion concentration compared to the reference air purged crucible with a value of 0.27 mol%. The probable cause for this will be further discussed at the end of the Results and Discussion section (Section 3.2.2). Similar to the oxide ion content in the air purged crucibles, the presence of steel in the **steel and nitrogen** purged crucible (C1–N_2_–S) compared to reference nitrogen purged (C3–N_2_-ref) results in an increased concentration of oxide ions up to 600 h as indicated in Fig. 4(b). However, after this point, the concentration is found to be lower compared to the case without steel (C3–N_2_-ref), indicating the consumption of these oxide ions in the presence of steel after reaching a concentration of 0.36 mol% at 600 h. This phenomenon could be linked to the consumption of oxide ions in the corrosion process once a certain level is reached within the salt, facilitating the formation of different corrosion products. Chromate ions are also one of these corrosion products, shows an increasing concentration at 600 h, as depicted in Fig. 4(c).

**Fig. 5:**
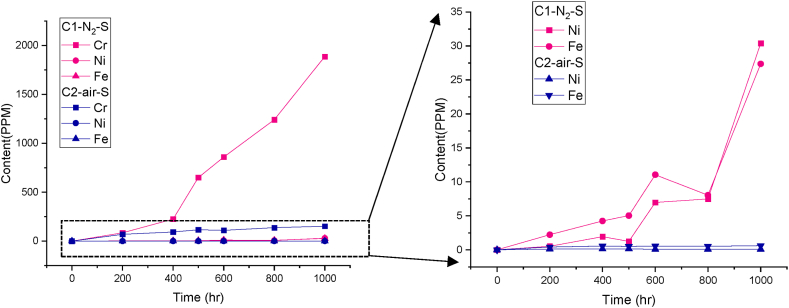
Concentration of metallic ions in salt exposed to nitrogen and air, as determined by atomic absorption spectrometry (AAS). The right graph presents an expanded view of the left graph's y-axis.

Following the observation of increased chromate content in the crucibles containing steel under both atmospheres, an additional analysis was conducted using AAS to identify the presence of other metallic species such as **iron, chromium, and nickel** in the same salt samples and also compared within the 2 atm, as depicted in Fig. 5.

In the **synthetic air-**purged crucible with steel (C2-air-S), the chromium content is found to be 152 ppm, while nickel and iron reached a lower value of less than 1 ppm, after 1000 h. However, in the **steel and nitrogen** purged crucible (C1–N_2_–S), the chromium concentration was 1885 ppm, while nickel and iron reached 27 and 30 ppm, respectively. The increased concentrations of these species within the salt under the nitrogen atmosphere further suggest the depletion of these elements from the steel. These elements are integral in the formation of a passive layer that serves to inhibit further corrosion, and their dissolution may suggest an increased corrosion rate. Additionally, out of the three analyzed species chromium exhibits the highest solubility in the salt melt, while nickel and iron have lower values in comparison. Interestingly, the concentration of nickel in the nitrogen-purged steel containing Solar Salt falls within the same range as that of iron, as illustrated in the right-side graph in Fig. 5, which indicates that diffusion of nickel from the steel into the salt may take place. This is further supported by the fact that the increase in nickel concentration in the Solar Salt cannot be solely attributed to the spallation of the corrosion layer, as the concentration of nickel in the steel is only 10–20 wt%, whereas Fe comprises over 60 wt% (refer to Table 1).

**Fig. 6:**
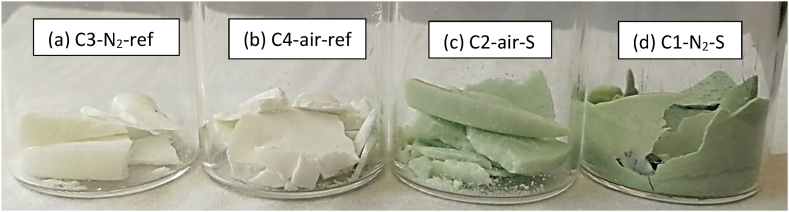
Solar Salt samples from different crucibles after 1000 h exposure at 600 °C in (a) nitrogen atmosphere (C3–N_2_-ref) (b) synthetic air atmosphere (C4-air-ref) (c) synthetic air and steel (C2-air-S) (d) nitrogen and steel (C1–N_2_–S).

In addition to the light green color resulting from chromate formation observed in the synthetic air and steel containing Solar Salt, as shown in Fig. 6(b)–a dark green color is evident in the nitrogen and steel containing Solar Salt samples, depicted in Fig. 6(d). This dark green color can be indicative for the formation of other dissolved transition metals such as iron, nickel or manganese. As expected, the reference crucibles shown in Fig. 6(a) and (b) do not exhibit any color change after exposure, as no steel samples were present inside. The impact of Solar Salt chemistry on its corrosivity will be further explored after analyzing the corrosion under these different atmospheres.

### 3.2 Stainless steel corrosion

#### 3.2.1 Mass loss by descaling

To investigate the influence of atmospheric conditions on the corrosion of austenitic steel, this section provides a detailed analysis of **mass loss by descaling** under two distinct atmospheres. The mass loss was calculated as per Equation (4). First, the mass loss in synthetic air is discussed, followed by the discussion of mass loss in a nitrogen atmosphere. Additionally, the mass loss of the two austenitic steel (AISI 316L and AISI 310) is also discussed and compared.

**Fig. 7:**
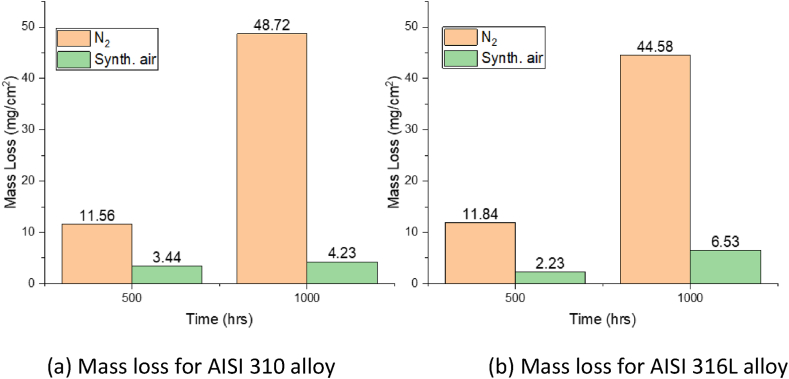
Area specific mass loss (mg/cm^2^) after 500 and 1000 h for (a) AISI 310 alloy and (b) AISI 316L in nitrogen and air atmosphere at 600 °C in Solar Salt.

In **synthetic air** atmosphere alloy AISI 310 shows a mass loss of 3.44/4.23 mg/cm^2^ after 500/1000 h respectively, as shown in Fig. 7(a). On the other hand, the mass loss for AISI 316L under the same condition is 2.23/6.53 mg/cm^2^ after 500/1000 h respectively, as shown in Fig. 7(b). In comparison between the two alloys, AISI 310 exhibits marginally superior corrosion resistance compared to AISI 316L after 1000 h in air, potentially attributed to the elevated chromium and nickel content present in AISI 310 (Table 1). These elements are recognized for their ability to generate protective metal oxide layers, mitigating corrosion effects. Furthermore, AISI 310 exhibits only a slight increase in corrosion mass loss after 500 h, suggesting the presence of a relatively stable corrosion-protective layer in comparison to AISI 316L.

In **nitrogen** atmosphere both AISI 310 and AISI 316L exhibit a very high mass loss compared to air purged steel samples, 11.56/48.72 mg/cm^2^ for AISI 310 (Fig. 7(a)), and 11.84/44.58 mg/cm^2^ for AISI 316L (Fig. 7(b)) after 500/1000 h, respectively. The substantial mass loss evident in the samples exposed to nitrogen signifies an elevated corrosion rate. These results underscore the corrosive nature of Solar Salt when subjected to a nitrogen-purged atmosphere, with the corrosion rate being approximately 10 times higher compared to exposure to air. These results are also consistent with the salt analysis, where higher concentrations of iron, chromium, and nickel were found in the nitrogen-purged crucible compared to the air-purged crucible. The increased corrosivity under nitrogen can be attributed to the high salt decomposition to produce high nitrite and oxide ions concentrations. Additionally, the role of different salt decomposition products (nitrate, nitrite, and oxide ions) in corrosion will be discussed at the end of the Results and Discussion section (Section 3.2.2).

#### 3.2.2 SEM-EDX analysis

**Fig. 8:**
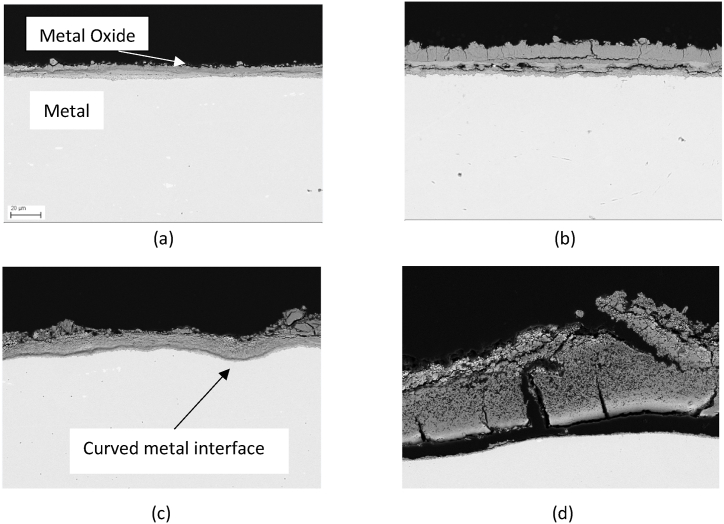
SEM micrograph of AISI 310 alloy cross-section after exposure to Solar Salt and air for a) 500 h, b) 1000 h, and after exposure to Solar Salt and nitrogen for c) 500 h and d) 1000 h.

To analyze the change in the corrosion layer thickness and structure, SEM analysis was performed on the cross section of the gold-sputtered steel samples after the corrosion test. The cross-sectional SEM micrographs in Fig. 8 illustrate the cross-section of AISI 310 alloy under air and nitrogen atmospheres after 500 and 1000 h, which exhibit similar characteristics to AISI 316L. For SEM micrographs of AISI 316L, please refer to Appendix A.1.

As depicted in Fig. 8(a) and (b), under the **synthetic air** atmosphere, the corrosion layer observed after 500 h exhibits a dense and non-porous structure. However, following 1000 h of exposure, the thickness of the corrosion layer increases, and the presence of both vertical and horizontal cracks along the steel surface becomes noticeable within the corrosion layer. The formation of these cracks can be attributed to a variety of factors, including intrinsic growth stress and thermally induced stresses arising from differences in the coefficient of thermal expansion (CTE) [27]. Additionally, the presence of loosely bonded particles on the outer corrosion layer further indicates the likelihood of spallation of the formed corrosion layer.

In contrast to the air atmosphere, the SEM micrographs in Fig. 8(c) and (d) reveal that the stainless-steel sample exposed to the **nitrogen** purged atmosphere exhibits a significantly thicker and highly porous corrosion layer, especially evident after 1000 h compared to the air purged atmosphere. After 1000 h, it was observed that the thick corrosion layer had detached from the underlying steel, suggesting a potential occurrence of increased spallation under the nitrogen atmosphere compared to air atmosphere. Furthermore, the porous nature of the oxide scale can also offer numerous pathways for the ingress of salt ions (nitrate, nitrite, or oxide ions), thereby exposing the underlying steel surface directly to molten salt and accelerating the corrosion process further. Additionally, the SEM micrographs, particularly Fig. 8(c), reveal the presence of a curved metal and oxide interface. The presence of a curved metal and oxide interface, indicates the occurrence of high compressive and tensile stresses [28]. These stresses can be attributed to factors such as differential thermal expansion between the metal and the corrosion layer, variations in metal oxide growth rates, or the buildup of internal stresses within the corrosion layer itself [28]. The observation of a curved interface provides additional evidence of the interplay between mechanical stresses and the corrosion layer growth.

For a comprehensive examination of the corrosion layer buildup on the same steel surface, the gold particles previously sputtered onto the steel surface were carefully traced in post-corrosion experiment with SEM cross-sections.

Interestingly, in the nitrogen atmosphere, extensive spallation and corrosion were observed, resulting in the absence of the gold layer in the SEM cross-section images for both alloys. Consequently, first, the gold particle tracing in synthetic air atmosphere steel samples is presented and discussed, along with the corresponding EDX elemental analysis. Subsequently, the SEM-EDX analysis in the nitrogen-purged atmosphere will be analyzed and discussed.

**Fig. 9:**
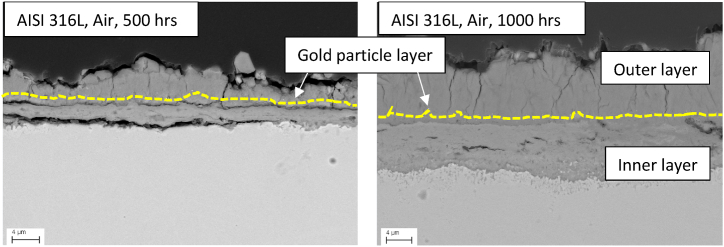
SEM images showing the sputtered gold particle layer for AISI 316L alloy after the corrosion experiment in Solar Salt exposed to air atmosphere after 500 and 1000 h at 600 °C. (For interpretation of the references to color in this figure legend, the reader is referred to the Web version of this article.)

**Fig. 10:**
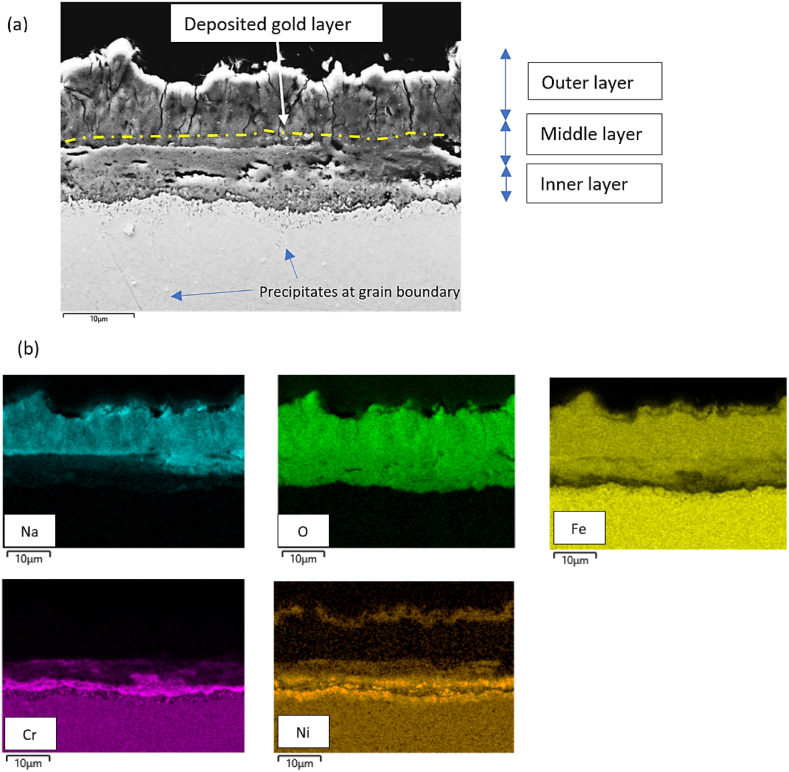
(a) Back scattered image (b) corresponding elemental distribution map of the corrosion layer and base metal for AISI 316L after 1000 h of exposure at 600 °C in Solar Salt exposed to air.

After the corrosion experiment, the gold particles were traced using line scans across the corrosion layer. An example of one such line scan is provided in Appendix A.2 for reference. The gold particles were found to exhibit high contrast in the Backscattered Electron (BSE) images, which were subsequently traced manually in the images. The gold particles traced after the corrosion experiment in **synthetic air** are illustrated as a yellow dotted line in Fig. 9 for AISI 316L steel (refer to Appendix A.3 for AISI 310). In the **synthetic air** atmosphere, the gold layer was observed in the middle of the corrosion layer for both alloys. Since gold particles were sputtered onto the metal surface before the corrosion experiment, their positions after the corrosion experiment can be used as markers to estimate the original dimensions of the metal surface. The presence of these particles in the middle of the corrosion layer indicates the buildup of the corrosion layer by both cationic diffusion towards the outer corrosion layer from the steel matrix i.e. metal cations and the anion counter diffusion from the salt to steel, as also explained by Sarrazin et al. [29]. To analyze the type of cation and anion diffusion across the corrosion layer an elemental distribution analysis was conducted on the same spot, as shown in Fig. 10(a). The corresponding elemental distribution map is presented in Fig. 10(b).

Based on the elemental distribution the formed corrosion layer can be divided into three different layers. First, the outer layer components are discussed followed by the middle layer and the innermost layer. In the outer layer (above the sputtered gold layer), the elements from the base metal like iron, chromium, and nickel are found indicating the outward diffusion of these elements. Additionally, sodium was also found in the outermost layer. The presence of sodium, oxygen, and iron in the outermost corrosion layer may suggests the formation of NaFeO_2_ or Na_4_Fe_2_O_5_, which is also reported by several other researchers [15,30]. The outer corrosion layer exhibits reduced protective capabilities for the inner corrosion layers, primarily attributed to the presence of prominent vertical cracks perpendicular to the steel surface. Additionally, the reduced protective capability of this layer can be attributed to its sodium ion-conducting nature. For example, NaFeO_2_ is commonly investigated for battery applications as a sodium ion-conducting membrane [31]. In the middle corrosion layer iron, oxygen and a trace of chromium are the dominant species, which shows the formation of chromium containing iron oxide layer. Below the gold-sputtered layer, the innermost layer shows chromium and oxygen indicating the formation of the chromium oxide layer, which is known to be protective in nature, further below this layer a high nickel-enriched zone can be observed. In addition to this, precipitates are also visible in the metal matrix at the grain boundary as indicated in Fig. 10(a). Soleimani et al. [32] found these to be chromium nitride precipitates along the grain boundary in 347H alloy at 600 °C corrosion test under air.

**Fig. 11:**
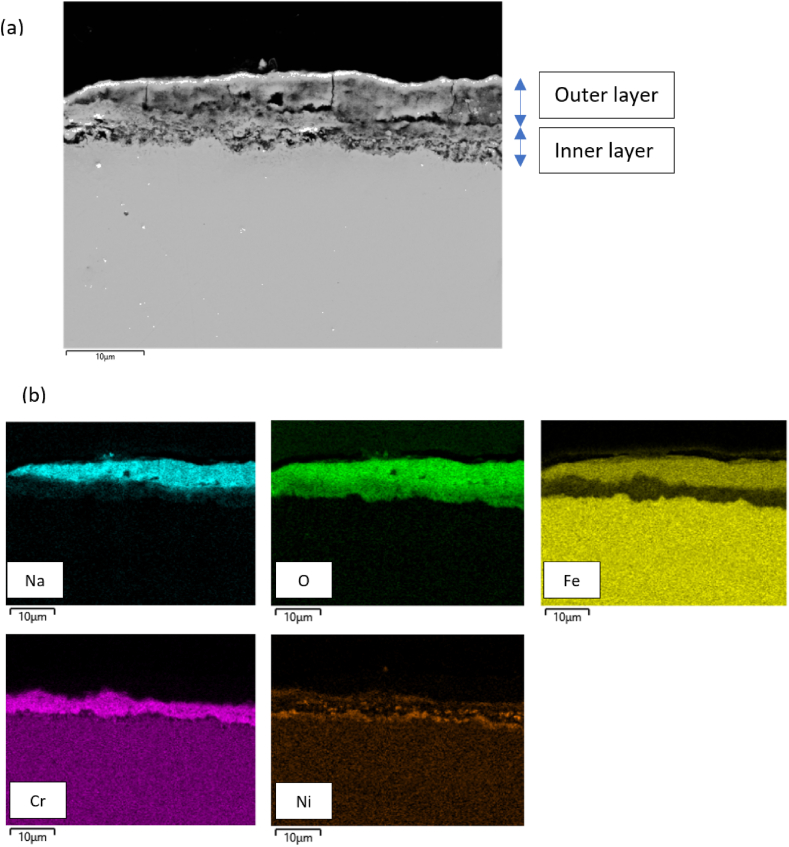
(a) Backscattered image and (b) corresponding elemental distribution map of the corrosion layer and base metal for AISI 316L after 1000 h of exposure at 600 °C in Solar Salt under nitrogen atmosphere.

In contrast to the air exposed Solar Salt and as shown in Fig. 11(a), the corrosion layer in the **nitrogen** exposed salt samples is thinner most likely due to excess spallation during the experiment. Unlike the air atmosphere, the nitrogen exposed steel samples reveal only two major layers, as analyzed from the elemental distribution map in Fig. 11(b). The outer layer contains iron, oxygen, and sodium, indicating the formation of sodium iron oxide layer i.e. NaFeO_2_ or Na_4_Fe_2_O_5_. The inner layer, however, mainly consists of chromium, oxygen, and a trace amount of nickel. An iron-chromium oxide layer is absent under nitrogen atmosphere. Due to the increased concentration of oxide ions resulting from salt decomposition under a nitrogen atmosphere, there is a preference for the formation of a sodium iron oxide outer layer. This phenomenon is supported by a study from Picard et al. [16], which showed that high concentrations of oxide ions can stabilize the formation of various sodium iron oxide compounds. Consequently, fewer amounts of hematite and magnetite are observed in the nitrogen atmosphere. The rapid formation of the outer sodium iron oxide layer due to the elevated oxide ion concentration diminishes its protective capability by establishing pathways for salt penetration to the base metal surface, the porous nature of the outer layer under nitrogen can be seen in Fig. 8(c) and (d). Consequently, this phenomenon can trigger the dissolution of the established protective chromium oxide layer, as indicated by the considerable chromate content detected in the IC analysis.

**Fig. 12:**
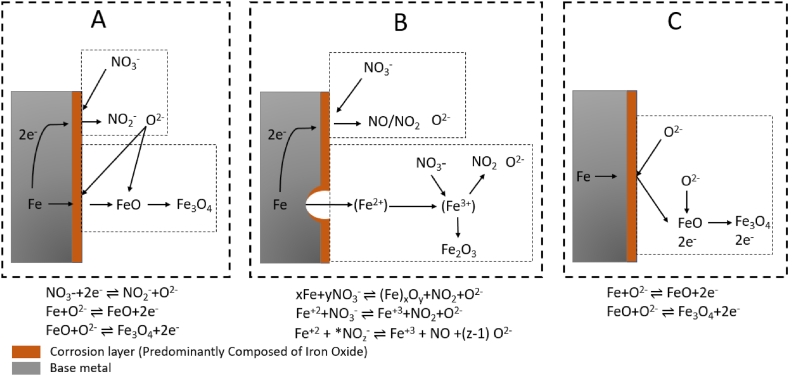
Three main corrosion processes from the literature indicating the interaction of nitrate, nitrite and oxide ions in nitrate molten salt, corrosion process A [34], process B [33,35], and process C [36].

Based on the results obtained in this study and the available literature, it is probable that the corrosion process in Solar Salt involves a complex interplay between salt chemistry and austenitic steel corrosion. Fig. 12 presents a visual representation of three different primary corrosion processes given in the literature [[33], [34], [35], [36]], demonstrating the probable involvement of nitrate, nitrite, and oxide ions in the corrosion of steel.

Corrosion processes shown in Fig. 12 A and 12 B illustrate the interaction between nitrate and nitrite ions. In **process A** nitrate is reduced to nitrite at the salt-steel interface, producing oxide ions. These newly generated oxide ions at the steel surface can subsequently oxidize the steel surface. In contrast, corrosion **process B** illustrates the reduction of nitrate at the steel interface to produce nitrogen oxide gases and oxide ions and further reaction of the formed corrosion layer of magnetite (Fe_3_O_4_) and nitrate ions to produce hematite (Fe_2_O_3_) and oxide ions. Additionally, following the same corrosion process Picard et al. [33] have also suggested the diffusion of these produced oxide ions inside the metal matrix to cause further corrosion. In Fig. 12C, **process C** illustrates the direct interaction of oxide ions produced from salt decomposition, leading to the formation of various corrosion products.

Based on the three corrosion processes outlined above and the results obtained in this study, the discussion will first focus on the **nitrate/nitrite and oxide ions** evolution in **synthetic air and steel compared to reference crucible without steel**, followed by an examination of the results in **nitrogen** atmosphere.

In the **synthetic air**-purged system, unlike corrosion processes A and B, the presence of austenitic steel (C2-air-S) in the synthetic air crucible did not result in a significant change in the nitrate/nitrite content compared to the condition without steel (C4-air-ref). The absence of a significant change in the nitrate/nitrite content in the air atmosphere suggests that the presence of steel in the air purged crucible does not strongly influence the formation or conversion of nitrate and nitrite ions, or the kinetics of the nitrite to nitrate oxidation reaction (reversed equation (1)) in presence of oxygen are quick enough to compensate for nitrite formation.

However, the observed elevation in **oxide ion** content for synthetic air and steel crucible compared to reference crucible without steel suggests that steel's presence does indeed contribute to the release or generation of oxide ions within the system. This aligns with the corrosion processes A and B, proposing the potential generation of oxide ions and NO_x_ (x is 1 or 2) gases during the initial interaction between the steel and nitrate ion. In addition, the consumption of these generated oxide ions in further corrosion was not observed, likely due to the presence of a passive and protective corrosion layer on the steel surface, or potentially due to the limited concentration of these ions.

In the **nitrogen**-purged system, the introduction of steel altered the **nitrate/nitrite** content, resulting in a slightly higher nitrite content. The difference in the nitrate and nitrite concentration in the nitrogen atmosphere with and without steel can be explained by the fact that the corrosion layer in the nitrogen atmosphere is highly porous and thick, this can be seen by the corrosion mass loss of approx. 4 mg/cm^2^ in air compared to a corrosion mass loss of 44 mg/cm^2^ in nitrogen after 1000 h. In the nitrogen atmosphere due to increased salt decomposition the oxide ion concentration increased significantly. The increased concentration of oxide ions from the salt decomposition under nitrogen can notably impact the corrosion process by increasing the formation of the corrosion layer, specifically sodium iron oxide. This finding is consistent with a previous study by Kugai et al. [37], where it was reported that the formation rate of sodium iron oxide, specifically α-NaFeO_2_, is influenced by the heat treatment atmosphere and the sodium source. For example, in an inert atmosphere such as nitrogen, pure α-NaFeO_2_ can be rapidly formed within a few hours of heating in the nitrate melt. The faster formation rate of sodium iron oxide under nitrogen can lead to increased defect density and hence porosity in the corrosion scale, compromising the integrity and protective properties of the formed layer, as evident in Fig. 8(c) and (d). The highly unstable and porous nature of the outer most corrosion layer allows the direct interaction of salt with the base steel surface. Therefore, the observed decrease in nitrate ion concentration and increase in nitrite ion concentration in the presence of steel suggests that nitrate ion reduction to nitrite ion at the salt-steel interface is possible, as shown in corrosion process A. In the air atmosphere, the differences in nitrate and nitrite concentrations may be more diluted or not as prominent due to the presence of a relatively passive corrosion layer, making it challenging to observe significant variations in the nitrate/nitrite content. Although corrosion process B also involves the consumption of nitrate ions to form oxide ions and NO_x_ gases, it cannot explain the increase in nitrite ion concentration and thus this reaction is deemed less likely to occur at the metal and salt interface. However, the consumption of nitrate ions with the already formed corrosion layer as shown in corrosion process B is more likely to occur at the already formed corrosion layer since an increase in oxide content was also observed after 200 h of exposure.

Furthermore, the observed increase in **oxide ions** concentration within the Solar Salt exposed to nitrogen-purged compared to air conditions can substantially increase the salt's basicity. The increased basicity, in turn, can facilitate a direct interaction between oxide ions and the steel, resulting in the formation of various corrosion products, including chromate and different iron oxides, as proposed by Bell et al. [38] in their investigation. Additionally, as demonstrated in corrosion process C, Baraka et al. [36] have indicated that an increase in salt basicity enhances the development of a passivation corrosion layer and have proposed the direct interaction of these oxide ions with the metal to form metal oxide. Moreover, Picard et al. [33] have also suggested a diffusion of these oxide ions from the melt into the base metal to form magnetite. Consequently, this chemical reaction can lead to a notable reduction in the overall concentration of these oxide ions within the molten salt, as also observed in the case of the nitrogen-purged system compared to steel and nitrogen purged system. The consumption of oxide ions started after the specific concentration of 0.36 mol% was reached at 600 h (as shown in Fig. 4(b)), indicating a change in the dominant corrosion mechanism, likely from A to C, between 600 and 1000 h, once this threshold concentration was attained.

In summary, the interaction between austenitic steel and the nitrate melt can result in an overall increase in the salt basicity (or amount of oxide ions). However, surpassing a certain concentration threshold can lead to their consumption by steel and subsequent slower increase of oxide ion concentrations in the salt. This critical concentration appears to have significant implications for the metallic components, as their consumption correlates with heightened corrosion as observed in the nitrogen purged systems.

Overall, the corrosion process in molten Solar Salt is a complex multistep process and can vary depending on the salt decomposition and its basicity. The integrity and stability of the initially formed corrosion layer on metal surfaces are crucial factors that can significantly influence the chemistry of Solar Salt in contact with austenitic steel. This formed outer corrosion layer of sodium iron oxide and iron chromium oxide acts as a protective barrier, by slowing down the further direct interaction between the salt and the metal. Its stability and integrity also determine the long-term performance and durability of the TES system. However, the stability of this corrosion layer can be affected by various factors other than the atmosphere and absolute temperature. Temperature fluctuations and turbulent flow of the Solar Salt within the TES system can also subject the corrosion layer to thermal stress and mechanical disturbances. These conditions can lead to the deterioration of the formed passive corrosion layer, compromising its protective properties. It is important to note that the specific corrosion products formed during the interaction between Solar Salt and the metal can vary. Various compounds, such as chromates and sodium iron oxides, may form depending on factors such as the composition of the Solar Salt and also the nature (e.g. metal surface finish, grain size, internal stress, etc.) of the metal surface.

## 4 Conclusion

This study explored the interplay between molten salt chemistry and steel corrosion in the two distinct purge-gas atmospheres synthetic air and nitrogen. This paper investigated the impact of varying atmospheres on nitrate/nitrite, oxide, and chromate content, along with the corrosion behavior of AISI 310 and AISI 316L stainless-steel alloys at 600 °C for 1000 h. The salt was analyzed using IC, AAS, and titration to determine the content of nitrate/nitrite, oxide ions, and the associated metal species (iron, chromium, and nickel) respectively. Additionally, the steel was also analyzed through mass loss by descaling and SEM-EDX cross-section analysis. This study also analyzed the alterations in salt chemistry with and without steel under different atmospheres. This leads to the following conclusions.

• **Influence of atmosphere on Solar Salt chemistry**: Nitrogen gas purging leads to higher Solar Salt decomposition compared to synthetic air atmosphere. The increased salt decomposition under nitrogen increases the oxide ions concentration in the Solar Salt approx. 7 times after 1000 h compared to air purged Solar Salt at 600 °C.
• **Influence of atmosphere on corrosion:** The Solar Salt decomposition and corrosion behavior of the steel is strongly influenced by the atmosphere. Corrosion in the nitrogen atmosphere is 10 times higher than in synthetic air after 1000 h according to descaling results. The formation of porous and less dense corrosion layer under nitrogen compared to air atmosphere supported the increased corrosivity of the Solar Salt. The porous nature of the formed corrosion scale facilitated the ingress of corrosive species and can accelerate the corrosion process further.
• **Impact of steel on Solar Salt chemistry:** The presence of austenitic steel in Solar Salt under air atmosphere increases the oxide ions concertation and does not influence the nitrate and nitrite ion concentrations. However, in the nitrogen atmosphere the overall oxide ion concentration in Solar Salt and austenitic steel increased slowly compared to the reference nitrogen purged atmosphere after reaching a certain threshold value, indicating oxide ion consumption by steel in the corrosion process. Additionally, the nitrate ions concertation was lower in the presence of austenitic steel, indicating direct interaction of nitrate ion with the steel surface through the porous corrosion layer to form nitrite ions.
• **Alloy Corrosion Resistance:** The mass loss measurements revealed a slightly better corrosion resistance of AISI 310 compared to AISI 316L in an air atmosphere, most likely due to the higher nickel (Ni) and chromium (Cr) content in the alloy. However, both alloys exhibited significant mass loss in the nitrogen atmosphere, underscoring the role of Solar Salt chemistry and its corrosive nature.
• **Gold particle tracing in air atmosphere:** The presence of gold particles in the middle of the corrosion layer indicates the buildup of the corrosion layer by both cationic diffusion towards the outer corrosion layer from the steel matrix i.e. metal cations (iron, chromium, and nickel) and the anions (oxide ions) counter diffusion from the salt to steel.
• **Significance of Oxide ions:** Oxide ions are crucial contributors to Solar Salt corrosion. Their increased concentration due to salt decomposition accelerates the formation of various corrosion products, such as sodium iron oxide scales on the steel surface and chromates formed in the molten salt. These products can impact the stability and integrity of the corrosion layer, affecting overall corrosion behavior.

Overall, this study highlights the crucial role of the atmosphere in controlling the corrosiveness of Solar Salt. It also highlights the relation between the formed corrosion layer and the salt chemistry. The absence of oxygen in the atmosphere significantly accelerated corrosion, leading to the formation of porous oxide scales and extensive mass loss. The findings underscore the importance of considering atmospheric conditions in the design and operation of solar TESs utilizing molten salts, as corrosion can compromise the integrity and longevity of materials.

## Data availability statement

Data will be made available on request.

## Additional information

No additional information is available for this paper.

## CRediT authorship contribution statement

**Sumit Kumar:** Writing – original draft, Validation, Methodology, Investigation, Conceptualization. **Andrea Hanke:** Writing – review & editing, Investigation. **Alexander Bonk:** Writing – review & editing, Validation, Supervision, Funding acquisition, Conceptualization. **Thomas Bauer:** Writing – review & editing, Project administration.

## Declaration of competing interest

The authors declare that they have no known competing financial interests or personal relationships that could have appeared to influence the work reported in this paper.

## Figures and Tables

**Fig. A.1:**
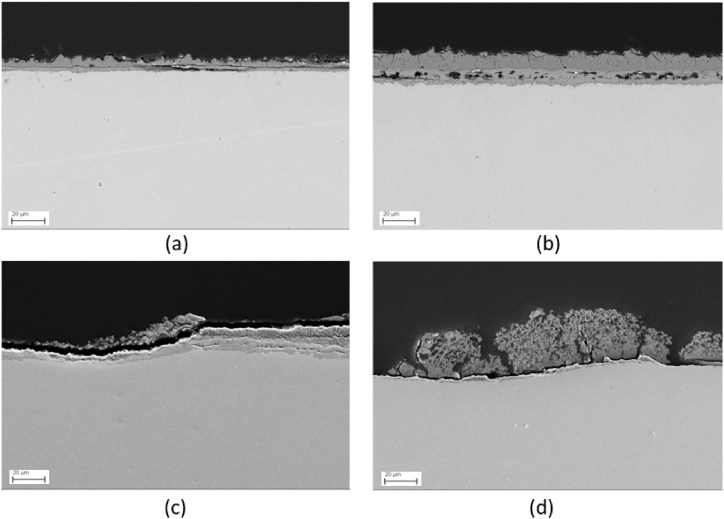
SEM micrograph of AISI 316L alloy cross-section after exposure to Solar Salt and air for a) 500 h, b) 1000 h and after exposure to Solar Salt and nitrogen for c) 500 h and d) 1000 h..

**Fig. A.2:**
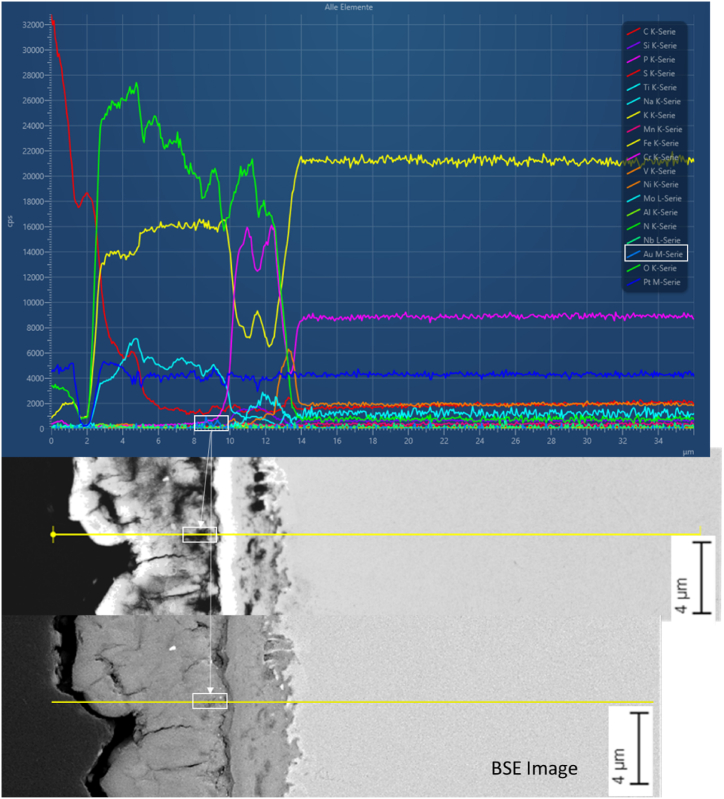
SEM micrograph of AISI 316L alloy cross-section after 1000 h of exposure to Solar Salt and air, illustrating gold particle tracing and a line scan across the corrosion layer..

**Fig. A.3:**
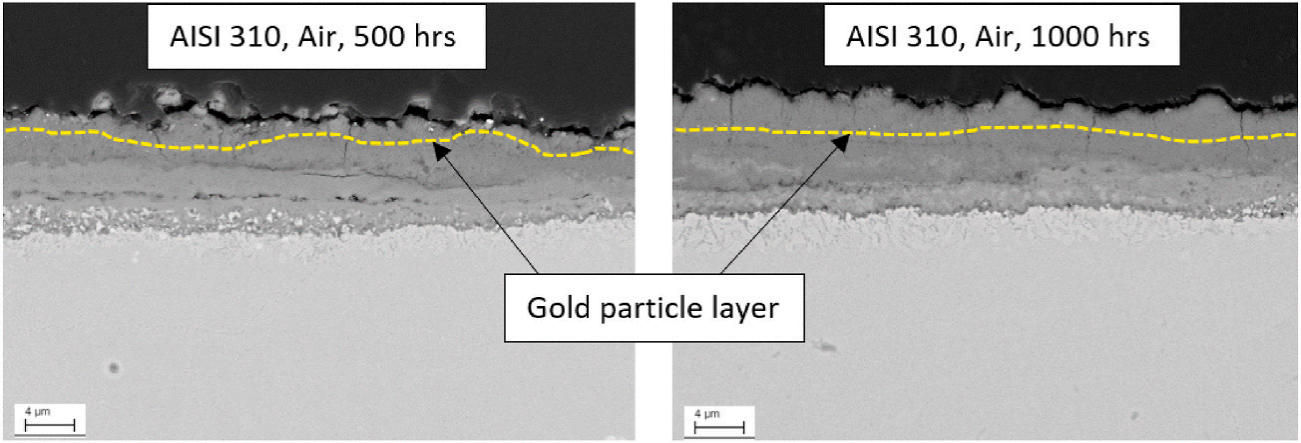
SEM images of AISI 310 alloy after the corrosion experiment in Solar Salt exposed to air atmosphere after 500 and 1000 h at 600 °C..
